# HiPro-AD: Sparse Trajectory Transformer for End-to-End Autonomous Driving with Hybrid Spatiotemporal Attention

**DOI:** 10.3390/s26010185

**Published:** 2025-12-26

**Authors:** Bing Chen, Gaopeng Wang, Jiandong Yang, Shaoliang Huang, Xinhe Qian, Bin Huang, Guanlun Guo

**Affiliations:** 1 Shandong Electric Power Engineering Consulting Institute Corp., Ltd., Jinan 250100, China; chenbing@spic.com.cn (B.C.); qianxinhe@spic.com.cn (X.Q.); 2 Hubei Longzhong Laboratory, Xiangyang 441106, China; whut1749981935@163.com; 3 School of Automotive Engineering, Wuhan University of Technology, Wuhan 430070, China; 4 Sdic Qinzhou Second Electric Power Co., Ltd., Qinzhou 535000, China; 10022685@sdic.com.cn (J.Y.); 18177969228@189.cn (S.H.)

**Keywords:** autonomous driving, bird’s eye view, trajectory planning

## Abstract

End-to-end (E2E) autonomous driving offers a promising alternative to traditional modular pipelines by mapping raw sensor data directly to vehicle controls, thereby mitigating error propagation. However, prevalent approaches largely rely on dense Bird’s-Eye-View (BEV) feature maps, which incur high computational overhead and necessitate complex post-processing for trajectory generation. To address these limitations, we propose HiPro-AD, a proposal-centric sparse E2E planning framework that fundamentally diverges from dense BEV paradigms. HiPro-AD integrates an efficiency-oriented IM-ResNet-34 encoder with a novel STFormer. This transformer dynamically fuses multi-view spatial features and historical temporal context via a proposal-anchored mechanism, focusing computation strictly on regions relevant to sparse trajectory proposals. Furthermore, trajectory selection is refined by a Pairwise Ranking Scorer, which identifies the optimal plan from diverse candidates based on relative quality. On the NAVSIM benchmark, HiPro-AD achieves a PDMS of 92.6 using only camera input, surpassing prior dense BEV and multimodal methods. On the closed-loop Bench2Drive benchmark, it attains a 37.31% success rate and a driving score of 65.48 with a latency of 67 ms, demonstrating real-time capability. These results validate the efficiency and robustness of our sparse paradigm in complex driving scenarios.

## 1. Introduction

End-to-end (E2E) autonomous driving, which directly regresses control commands or future trajectories from raw sensor inputs, has emerged as a promising alternative to traditional modular pipelines. By eliminating hand-crafted intermediate representations and heuristic rules, E2E systems mitigate error propagation and significantly improve adaptability in complex urban scenarios [1,2,3]. Meanwhile, robust control methods have tackled safety challenges, such as secure observer-based control under non-Gaussian noises [4] and cloud-based collision avoidance with token bucket shapers [5]. Recent advances, such as UniAD [6], VAD [7], and DiffusionDrive [8], have achieved impressive closed-loop performance by adopting dense Bird’s-Eye-View (BEV) feature grids as the unified scene representation. These dense BEV encoders enable rich spatial reasoning and seamless integration of multi-view imagery through learned view transformation.

In recent years, the research landscape has evolved into several distinct learning paradigms. As summarized in Table 1, these generally include Imitation Learning (IL), Reinforcement Learning (RL), Knowledge Distillation, and World Models. Among these, Imitation Learning has become the dominant approach, with representative methods such as UniAD [6] and VAD [7] achieving impressive performance by cloning expert behaviors from large-scale datasets. Reinforcement Learning algorithms (e.g., PPO [9], SAC [10]) offer potential for long-horizon planning through trial-and-error but face challenges in sample efficiency. Knowledge Distillation methods (e.g., Roach [11]) and World Models (e.g., MILE [12]) further explore privileged teacher guidance and self-supervised dynamics modeling, respectively.

Despite their success, dense BEV-based paradigms suffer from fundamental limitations that hinder real-world deployment. First, constructing high-resolution dense BEV grids incurs quadratic computational complexity with respect to spatial resolution, resulting in excessive memory footprint and latency. Second, dense representations indiscriminately process large irrelevant background regions, diluting trajectory-specific gradients and degrading planning performance in safety-critical yet rare scenarios (e.g., occlusion, cut-ins, and unprotected turns). Moreover, most existing methods still rely on imitation learning with massive non-reactive simulation rollouts to stabilize training, further amplifying computational burden.

In response to the above issues, the HiPro-AD method proposed in this paper represents a fundamental shift in principle. It innovatively adopts a proposal-centric sparse paradigm, where the core idea is to treat the planning task—namely, generating future trajectories—as the central task in the entire perception and decision-making process. Specifically, HiPro-AD first extracts key features from multi-view images using a lightweight and efficient IM-ResNet-34 scene encoder. This encoder utilizes depthwise separable convolutions and ECA channel attention mechanisms to significantly reduce computational overhead while maintaining feature quality. Based on the ego vehicle state and learnable embeddings, HiPro-AD then initializes a set of sparse trajectory proposals in BEV space, which serve as the queries for subsequent reasoning.

Next, the STFormer module bypasses the intermediate step of dense BEV grid construction and keeps these sparse trajectory proposals as the core representation throughout the network. Through an iterative process, each encoder layer applies proposal-anchored deformable self-attention to model interactions within and between trajectories, followed by a Temporal Fusion Encoder (TFE) that leverages a BEV memory bank and temporal cross-attention to integrate historical proposal features, and finally spatial cross-attention that selectively fuses the most relevant information from multi-view feature maps. This “on-demand” feature fusion approach greatly improves computational efficiency and planning awareness compared to building a complete BEV, while the explicit temporal fusion ensures robustness in occluded or highly dynamic scenarios.

To further improve trajectory quality, HiPro-AD employs a Scorer based on pairwise ranking loss. The model learns to compare different proposals relative to each other, allowing for more precise selection of the optimal planning solution. Additionally, by using proposal-centric auxiliary prediction tasks, the model not only plans trajectories but also understands the potential risks associated with each one, enhancing decision-making interpretability.

Our contributions can be summarized as follows:

1. Efficient Feature Extraction Network: An efficient IM-ResNet-34 backbone incorporating depthwise separable convolutions and Efficient Channel Attention (ECA), which substantially reduces computational overhead while maintaining feature quality.
2. STFormer, a novel sparse transformer that iteratively refines trajectory proposals via proposal-anchored deformable self-attention, explicit temporal fusion from a BEV memory bank, and geometry-constrained spatial cross-attention. Combined with a Top-k multi-modal regression loss, STFormer eliminates the need for dense intermediate representations and achieves superior training stability without closed-loop rollouts.
3. A lightweight pairwise ranking scorer that directly optimizes relative proposal quality using simulation-derived composite metrics, enabling precise selection of the optimal trajectory from diverse high-quality candidates and enhancing interpretability.

## 2. Related Works

### 2.1. End-to-End Autonomous Driving

Compared to discrete module-based driving systems, end-to-end autonomous driving opens up a new technical approach that learns policies directly from vehicle states and sensor data of the surrounding environment. By bypassing intermediate components, it eliminates potential information bottlenecks and accumulated errors, allowing the network to continuously optimize towards a final goal similar to human drivers. This concept can be traced back to the late 1980s with Carnegie Mellon University’s ALVINN [1] project, which first demonstrated the feasibility of predicting steering directly from images. In 2016, NVIDIA’s PilotNet [2] model achieved success in real-world driving through imitation learning, marking a substantial advancement in this approach. To capture the temporal dynamics in driving, subsequent studies, such as the FCN-LSTM [16] model, combined convolutional networks (FCN) with long short-term memory networks (LSTM), enabling video-based decision-making. In recent years, the performance boundaries of end-to-end models have continued to expand with the introduction of Transformer [17] architectures and multimodal data, such as LiDAR.

Currently, end-to-end autonomous driving relies on two main paradigms: imitation learning and reinforcement learning. Imitation learning [18] is a form of supervised learning that trains models to replicate human driver behavior by learning from large datasets of “sensor data-expert actions” pairs. PilotNet is a classic example of this approach. To overcome the limitations of simple imitation learning in scene understanding, subsequent research incorporated recurrent neural networks (RNNs) to handle temporal information and used multi-task learning to enhance environmental perception. Conditional imitation learning (CIL) [19] guides decision-making with high-level instructions (e.g., “turn left”). Knowledge distillation techniques (such as the LBC and Roach models) use a teacher model with an “omniscient view” to guide the student model, effectively improving performance [11]. Currently, Transformer-based multimodal fusion models (e.g., Transfuser [13]) and methods incorporating BEV representations have become research hotspots, significantly enhancing the robustness of systems in complex urban environments.

Reinforcement learning [20], on the other hand, takes a different approach by enabling the agent to autonomously learn optimal driving strategies through trial and error interactions with the environment, guided by reward signals. Its application has expanded from simple lane-keeping tasks to more complex ones, such as intersection navigation and multi-agent collaboration. Algorithms like DDPG [21] and A3C [22] are commonly used in this domain. However, reinforcement learning faces three major challenges: difficulty in designing reward functions, low sample efficiency, and the simulation-to-reality gap (Sim-to-Real Gap). To address these issues, researchers have explored hierarchical reinforcement learning and strategies that combine imitation learning and reinforcement learning (e.g., CIRL [23]) to improve learning efficiency and policy quality.

### 2.2. Attention Mechanism

As a core technology in computer vision, the attention mechanism mimics the selective attention characteristic of the human visual system, enabling neural networks to dynamically adjust the weight of information processing. This allows for efficient perception in complex scenarios. In end-to-end autonomous driving planning tasks, the attention mechanism is particularly important as it helps the model focus on the environmental information most relevant to trajectory generation, improving both planning accuracy and robustness. In the domain of channel attention, SENet [24] pioneered the squeeze-and-excitation (SE) structure, which models channel dependencies through global average pooling and fully connected layers, providing a new paradigm for feature recalibration. Subsequent research has continuously expanded on this foundation: GSoP-Net [25] introduced second-order statistics to enhance feature representation, while ECANet [26] used 1D convolutions to optimize cross-channel interactions. These advancements have collectively pushed channel attention from basic statistical modeling to more refined relational modeling.

The development of spatial attention mechanisms follows an evolutionary path from explicit localization to global modeling. Early works, such as STN [27], performed explicit region selection through spatial transformations, while deformable convolutions improved flexibility through adaptive sampling. A breakthrough came with the introduction of self-attention mechanisms: the Non-Local [28] network pioneered a new paradigm for long-range dependency modeling, and Vision Transformer [29] revolutionized the traditional approach by converting images into sequential inputs. Later, Swin Transformer [30] significantly enhanced computational efficiency while maintaining global modeling capability through hierarchical design and a sliding window mechanism.

Hybrid attention mechanisms have expanded the application boundaries by integrating multiple dimensions. The CBAM [31] module achieved channel-space collaborative optimization through serial stacking, while BAM [32] enhanced feature selection ability using parallel paths. Triplet Attention [33] emphasizes cross-dimensional interaction, and Coordinate Attention improves spatial perception by using positional encoding. These methods have demonstrated remarkable advantages in complex scene understanding tasks.

To provide a clearer perspective on these developments, Table 2 systematically compares these mechanisms and delineates the rationale for their specific adoption in the HiPro-AD framework.

## 3. Methods

**Figure 1:**
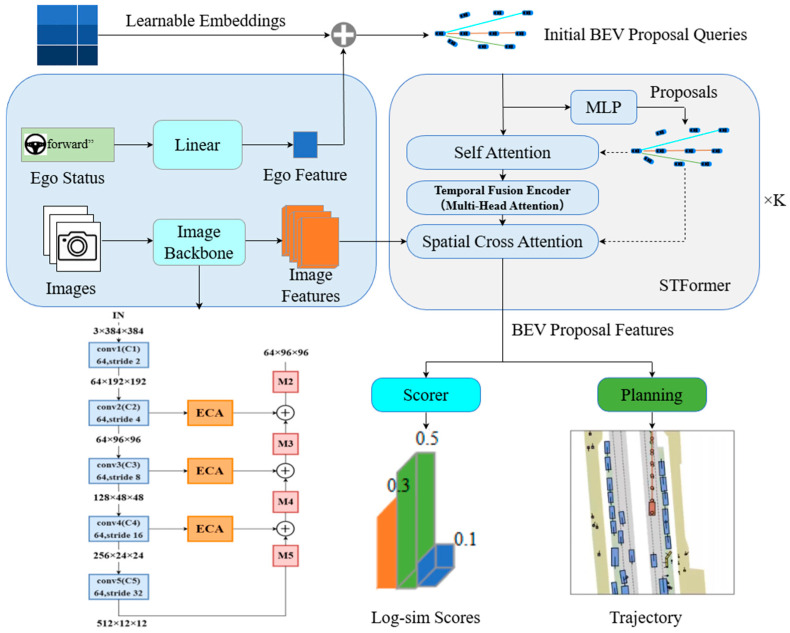
Overall Framework. HiPro-AD adopts a proposal-centric sparse paradigm. The Scene Encoder extracts features from multi-view images and ego status. STFormer then iteratively refines these proposals via proposal-anchored self-attention, a Temporal Fusion Encoder (TFE), and spatial cross-attention, yielding BEV proposal features. Finally, a ranking-based Scorer evaluates the proposals to select the optimal trajectory.

The overall architecture of our method, HiPro-AD, is illustrated in Figure 1. HiPro-AD consists of three main parts: (i) a Scene Encoder, which processes multi-view input images and the ego vehicle state to extract image features and an ego feature; (ii) an STFormer, which takes the initial BEV proposal queries and iteratively refines them using deformable self-attention, a Temporal Fusion Encoder (TFE) operating on a BEV memory bank, and spatial cross-attention to multi-view image features, yielding BEV proposal features; and (iii) two lightweight heads, a Scorer and a Planning module, which consume the BEV proposal features, where the Scorer predicts log-sim scores for all proposals and the Planning head decodes the highest-scoring proposal into the final trajectory.

### 3.1. Scene Encoder

Our method takes two types of input: multi-view images and the vehicle’s state. In HiPro-AD, camera images from N views of the vehicle first pass through a shared image encoder for 2D feature extraction.Facilitated by the strict temporal synchronization and precise sensor calibration inherent to the NAVSIM [34] dataset, these multi-view inputs are processed simultaneously to ensure spatial consistency. The encoder consists of two parts: the backbone and the neck. The backbone uses an improved ResNet-34 architecture, followed by the neck, which employs a Feature Pyramid Network (FPN) to integrate features from different scales into a unified channel dimension, resulting in multi-view feature maps. The multi-view feature map I is represented as:

(1) $$I\in R^{N\times C\times H\times W}$$

where N is the number of views, C is the number of channels, and H and W represent the spatial dimensions of the feature map.

Meanwhile, the vehicle’s state (such as current speed, acceleration, and future commands) is normalized and then encoded through a linear layer into a vector $E\in R^{1\times C}$, which has the same dimensionality as the visual features. These visual and state features are later fused and processed together in the proposal center module for subsequent reasoning.

To reduce computation and model parameters while improving real-time performance, we replace the first 3 × 3 convolution in each standard residual block of ResNet-34 with a depthwise separable convolution of the same kernel size, resulting in the improved IM-ResNet-34. This replacement significantly reduces the number of multiply accumulate operations and model parameters without changing the receptive field, making it more efficient for detection tasks while retaining the ability to model local textures.

In the neck, we construct a feature pyramid to enhance spatial information representation. Specifically, the deep features M5, M4, and M3 are upsampled and then fused with shallow features C4, C3, and C2, which have the same size and channel dimensions. Before each upsampling step, a 3 × 3 deformable convolution is applied to adjust the feature channels of M5, M4, and M3 to 256, 128, and 64, respectively, ensuring the feature dimensions match.

To further enhance the model’s ability to express semantic information, a channel attention ECA module is introduced before the fusion of deep and shallow feature layers. This enables the network to automatically strengthen its focus on target features while reducing attention to background information.

**Figure 2:**
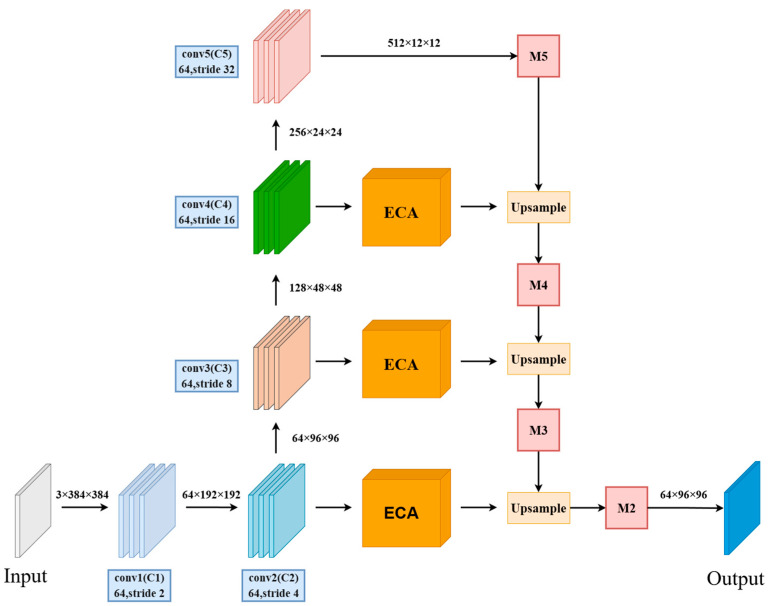
IM-ResNet-34 Architecture. The backbone employs depthwise separable convolutions to reduce computational cost. Deep features are upsampled and fused with shallow features via a Feature Pyramid Network (FPN), which incorporates ECA channel attention to enhance semantic representation.

Figure 2 illustrates the structure of the improved IM-ResNet-34 network. Each layer in the ResNet-34 network consists of a standard residual module with two 3 × 3 convolutional layers. In the improved IM-ResNet-34 backbone, the first standard 3 × 3 convolution in each residual module is replaced with a depthwise separable convolution of the same kernel size, reducing model parameters and computation. To enhance the model’s spatial information representation, the upsampled deep feature maps M5, M4, and M3 are fused with shallow features C4, C3, and C2 of the same size and channels to form a feature pyramid network. Before each upsampling, 3 × 3 deformable convolutions are applied to adjust the feature channels of M5, M4, and M3 to 256, 128, and 64, respectively. Additionally, to improve the model’s semantic information expression, a channel attention ECA module is applied to the shallow feature layers before fusion, emphasizing the target features.

### 3.2. STFormer

Inspired by the iterative proposal-centric paradigm of iPad [35], we propose STFormer, a sparse, end-to-end trajectory planning network. STFormer places proposals (i.e., candidate trajectories) at the core of feature extraction, iteratively refining them using multimodal sensor data and temporal history. Unlike traditional methods relying on dense BEV grids, our sparse proposal-centric paradigm significantly enhances computational efficiency. The proposal extraction and refinement are integrated into a unified encoder layer, stacked *K* times. Each encoder layer consists of three sub-modules applied in sequence: proposal-anchored deformable self-attention, a Temporal Fusion Encoder (TFE) operating on a BEV memory bank, and spatial cross-attention to multi-view image features.

STFormer adopts a proposal-centric sparse representation, where each proposal represents a complete future trajectory sequence, mathematically denoted as $P_{k}\in R^{N\times T\times 3}$, with N being the number of proposals, and T the time steps. Each time step’s state contains two-dimensional position coordinates $\left(x,y\right)$. This representation significantly reduces computational complexity while maintaining rich spatiotemporal information. The initialization of proposals is based on the vehicle’s current state, such as speed, acceleration, and future commands. This information is encoded as an ego feature $E\in R^{1\times C}$ and summed with learnable positional embeddings through a linear layer to generate the initial BEV proposal queries $Q_{0}$. In each iteration k = 0, 1,…, k−1, the current BEV proposal queries $Q_{k}\in R^{N\times T\times C}$ are directly mapped into a proposal sequence $P_{k}$ through a Multi-Layer Perceptron (MLP) network, represented as:$\;P_{k}=MLP\left(Q_{k}\right)$. Subsequently, we apply a proposal-anchored Deformable Self-Attention (SA) to the queries. By utilizing the predicted proposal positions from the MLP as spatial anchors, this mechanism effectively captures the temporal dependencies within trajectories and the interactions among different proposals. Specifically, for each query $Q_{k}^{n,t}$, a linear projection layer predicts sampling offsets Δp relative to the anchor point $P_{k}^{n,t}(x,y)$. The attention weights are simultaneously learned to aggregate features from these sampled locations, allowing the model to adaptively attend to spatiotemporally relevant proposals within the sparse set. The self-attention process is formulated as:

(2) $$SA(Q_{k}^{n,t},Q_{k})=\mathrm{DeformAttn}(Q_{k}^{n,t},P_{k}^{n,t}(x,y),Q_{k}),$$

where $Q_{k}^{n,t}$ represents the query for the *n*-th proposal at time step *t*, and $P_{k}^{n,t}(x,y)$ serves as the reference point for the deformable attention operation over the query set $Q_{k}$.

**Figure 3:**
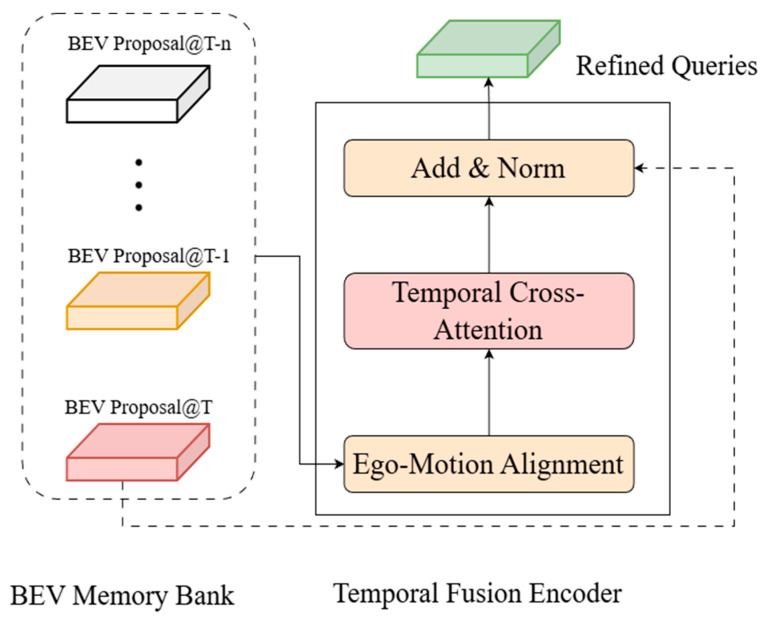
Temporal Fusion Encoder. Historical sparse proposals from the memory bank are aligned via Ego-Motion Alignment and fused with current queries using Temporal Cross-Attention to incorporate spatiotemporal context.

To explicitly exploit temporal context, as illustrated in Figure 3, we maintain a Memory Bank storing the refined proposal features $M_{t-1}$ from the previous frame. Before fusion, the historical proposals are aligned to the current ego-coordinate system via the Ego-Motion Alignment module, which leverages high-precision localization data to accurately compensate for vehicle movement. Mathematically, we apply a rigid transformation matrix $T_{t-1\to t}\in R^{3\times 3}$, derived from the ego-vehicle’s odometer, to transform the historical coordinates $P_{t-1}$ into the current ego-coordinate system. Unlike spatial features which are dense, our historical context consists of sparse proposal vectors. Therefore, instead of deformable attention, we employ Multi-Head Cross-Attention (MHCA) to fuse the current queries with historical features. This allows the current proposal to “attend” to relevant historical trajectories:

(3) $$TCA(Q_{k},M_{t-1}^{\prime })=MHCA(Q_{k},M_{t-1}^{\prime },M_{t-1}^{\prime })$$

Here, $M_{t-1}^{\prime }$ denotes the motion-aligned historical proposal features.

**Figure 4:**
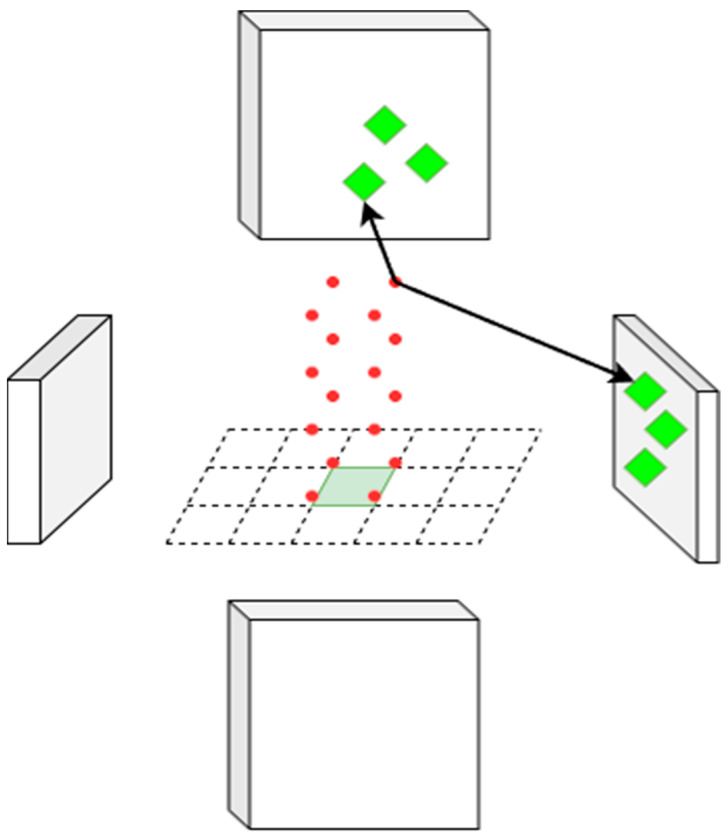
Spatial Cross-Attention. The process consists of four steps: (1) Proposal Anchoring: The green square on the central grid represents a hypothetical proposal anchor (a predicted vehicle position) in the BEV space. (2) 3D Sampling: To capture vertical geometric information beyond the ground plane, we lift the proposal corners into pillars and sample 3D reference points, which are depicted as floating red dots. For each proposal corner, we uniformly sample $N_{ref}$ points along the z-axis (height) to capture 3D geometric information. (3) Projection: These 3D points are projected onto the surrounding blocks, representing the multi-view image feature maps captured by different cameras. (4) Feature Aggregation: The Green diamonds indicate valid projections on “Hit Views,” where deformable attention aggregates visual features to refine the trajectory.

Finally, the temporally enhanced queries fuse multi-view visual features via Spatial Cross-Attention (SCA), as shown in Figure 4.

Spatial cross-attention uses the predicted proposals to compute attention weights between the proposal queries and the image features I. For each proposed pose, the vehicle’s four corner points are computed as BEV anchors, which encode the vehicle size and planned heading. Reference points sampled along vertical pillars lifted from these anchors are projected onto the 2D image planes, and deformable attention aggregates image features around the projected points. For a given BEV query, the projected 2D points fall only within a subset of camera views and may miss others. For each BEV query $Q_{k}^{n,t}$, we use the four proposal corners as anchors to aggregate features from the relevant camera views, as in Equation (4).

(4) $$\;SCA\left(Q_{k}^{n,t},I\right)=\frac{1}{\left|V_{hit}\right|}\sum_{i\in V_{hit}}\sum_{j=1}^{4}\sum_{z=1}^{N_{ref}}DeformAtt\left(Q_{k}^{n,t},P\left(P_{k}^{n,t},i,j,z\right),I_{i}\right)$$

Here, the projection function Ρ maps 3D reference points onto the image plane using camera intrinsic and extrinsic parameters, and $V_{hit}$ denotes the set of camera views that receive valid projections. This design ensures that the model simultaneously enforces temporal consistency and geometric constraints.

We design STFormer with shared weights across iterations. While the standard Minimum-over-N (MoN) loss is commonly used for supervision, it suffers from sparse gradients by optimizing only the single best proposal, leading to training instability. To address this, we propose a Top-k Multi-Modal Loss, which optimizes the subset of proposals closest to the ground truth. This approach balances sufficient gradient flow for rapid convergence with the preservation of multimodal diversity. The formulation is:

(5) $$L_{proposal}=\sum_{k=0}^{K-1}\;\lambda_{k}\frac{1}{\left|S_{top}\right|}\sum_{n\in S_{top}}\;∥P_{k}^{n}-\hat{P}{∥}_{1}$$

where $P_{k}^{n}$ is the n-th proposal trajectory at iteration k, $\hat{P}$ is the expert trajectory, and $\lambda_{k}$ is the iteration discount factor. $S_{top}$ represents the set of indices of the top-*M* proposals that have the smallest Euclidean distance to the expert trajectory $\hat{P}$, and $\left|S_{top}\right|$ = *M*. By setting 1 < *M* < *N*, the model learns to refine multiple high-quality hypotheses simultaneously without suppressing multimodal behaviors.

### 3.3. Scorer

**Figure 5:**
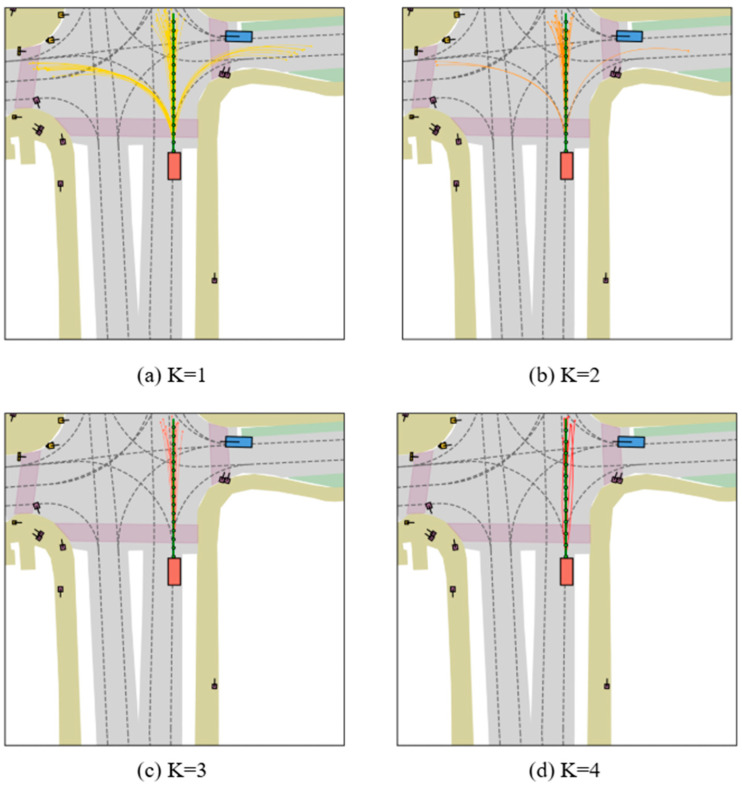
Visualization of trajectory generation and evolution. The green line represents the ground-truth trajectory. (**a**) The first iteration generates 64 initial proposals from learnable embeddings, showing a divergent distribution to maximize search space coverage. (**b**,**c**) The proposals undergo progressive geometric refinement through intermediate STFormer layers. (**d**) By the fourth iteration, the proposals converge into a compact set of smooth trajectories aligned with the lane topology, serving as the optimized candidate input for the Scorer.

The Scorer evaluates a set of candidate trajectories generated via a learnable “Query-to-Curve” mechanism. Instead of relying on fixed anchors, this framework evolves sparse proposals from a shared origin—the ego vehicle’s current position—to ensure coverage of potential driving intents. The process begins with an Initialization Phase, where the ego vehicle’s kinematic state is fused with 64 learnable positional embeddings. These embeddings serve as seeds at the origin and are decoded into 64 initial proposals via a Multi-Layer Perceptron. As depicted in Figure 5a, the generated curves at the first iteration exhibit a dispersed, fan-shaped spatial distribution, designed to cover a broad search space of potential driving modes such as lane keeping and turning.

Subsequently, the proposals enter an Iterative Refinement Phase through four layers of the STFormer. Guided by the Top-k Multi-Modal Loss, the model dynamically interacts with scene context to update the spatial geometry of the candidates. Intermediate refinements are observable in Figure 5b,c. Finally, Figure 5d demonstrates the outcome after the fourth iteration, where the initially dispersed proposals converge into a compact set of trajectories. These refined candidates are physically plausible and aligned with the map topology, constituting the final input set for the Scorer’s ranking operation.

To select the optimal plan from the set of trajectories obtained after iterative refinement, we design a scorer trained with a pairwise ranking loss. Rather than predicting an absolute score for each proposal independently, the scorer directly optimizes the relative ordering among proposals, enabling more accurate identification of the best trajectory.

Concretely, the workflow proceeds as follows: STFormer outputs the final proposal features $Q_{K}^{n}\in R^{T\times C}$, which have already been enriched by both spatial cross-attention and the Temporal Fusion Encoder. The Scorer takes these temporally fused features as input. We apply max pooling along the temporal dimension *T* of each proposal feature $Q_{K}^{n}$ to aggregate sequence information and obtain a global representation. A lightweight multilayer perceptron (MLP) then maps this global representation to a scalar score $s_{n}$. The scores of all *N* proposals form a vector $S\in R^{N}$.

We train the scorer with a pairwise ranking loss, which encourages the model to assign higher relative scores to truly better proposals. For each pair (*i*,*j*), the loss is:

(6) $$L_{Pairwise}=-\frac{1}{\left|P\right|}\sum_{\left(i,j\right)\in P}log\left(\sigma \left(s_{i}-s_{j}\right)\right)$$

P is the set of pairs $\left(i,j\right)$ in which proposal i is preferred to proposal j.$\;\sigma \left(\cdot \right)$ is the sigmoid function, mapping the score difference $s_{i}-s_{j}$ to a probability—the confidence that proposal i is better than proposal j.

Ground-truth labels for proposal preference follow NAVSIM’s composite evaluation. For each generated proposal, we execute a non-reactive log-replay simulation to measure five key sub-metrics: No At-Fault Collision (NC), Drivable Area Compliance (DAC), Ego Progress (EP), Time-to-Collision (TTC), and Comfort (Comf). These metrics are aggregated into a scalar Ground-Truth Score using the weighted formula defined by the PDM-Score:

(7) $$\hat{S}=NC\times DAC\times \frac{5\times EP+5\times TTC+2\times Comf}{12}$$

During training, the pairwise ranking loss (Equation (6)) utilizes the difference in these target quality scores $s_{i}-s_{j}$ to determine the relative preference, thereby aligning the model’s selection criteria with the comprehensive driving quality defined by NAVSIM.

## 4. Experiments

To evaluate our method, we conducted experiments on the open-loop NAVSIM dataset. The model architecture and hyperparameters are summarized in Table 3. Training was performed for 20 epochs on two NVIDIA RTX 4090 GPUs (NVIDIA Corporation, Santa Clara, CA, USA) using the Adam optimizer (Meta Platforms, Inc., Menlo Park, CA, USA) with a learning rate of 1 × 10^−4^. For efficiency, we used downsampled images from the front, left, right, and rear camera views as inputs.

### 4.1. NAVSIM Benchmark

Open-loop Evaluation on NAVSIM. We evaluate our method on the open-loop NAVSIM [34] benchmark, a data-driven, non-reactive simulation and evaluation platform designed for end-to-end planning. Built on real driving data from nuPlan, NAVSIM filters out many trivial scenarios (e.g., steady straight-line driving) and retains more challenging cases to enable more informative assessment. Its key feature is non-reactive simulation: during evaluation, other traffic participants do not respond to the ego vehicle’s planned trajectory but strictly replay their logged motions. This preserves the richness of real data while allowing simulation to compute composite metrics that approximate closed-loop testing. We use the official NAVTRAIN and NAVTEST splits, containing 103K and 12K samples, respectively, for training and evaluation.

NAVSIM introduces a set of closed-loop-oriented metrics to evaluate open-loop simulation. The submetric scores align with our training submetrics, and NAVSIM further defines the Planning Decision Metric Score (PDMS) as:

(8) $$PDMS=NC\times DAC\times \frac{5\times EP+5\times TTC+2\times Comf}{12}$$

where submetrics are computed over a 4-s non-reactive simulation window. A kinematic bicycle model controlled by an LQR controller tracks the planned trajectory to simulate the ego vehicle at 10 Hz. These submetrics are computed from the simulated ego trajectory, the logged trajectories of other agents, and the map.

To provide a rigorous quantification of the planning performance, we detail the mathematical formulations of the Key Performance Indicators (KPIs) used in the PDMS.

No At-Fault Collision (NC): A discrete safety penalty based on collision types. Collisions with vehicles, pedestrians, or bicycles result in a zero score. The score is computed as:

(9) $$NC=\left\{\begin{array}{lll} 1 & if\;no\;collision & \\ 0.5 & if\;non-at-fault\;collision\;(e.g.,\;static\;object) & \\ 0 & if\;at-fault\;collision\;(road\;users) & \end{array}\right.$$

Drivable Area Compliance (DAC): A binary indicator ensuring the ego vehicle remains within the road boundaries. Let $S_{t}$ be the set of ego vehicle corner coordinates at time t, and R be the drivable area polygon. Any corner leaving the drivable area results in a score of 0. The score is given by:

(10) $$DAC=\left\{\begin{array}{lll} 1 & if\;\forall t\in [0,T],\forall p\in S_{t},p\in R & \\ 0 & otherwise & \end{array}\right.$$

Ego Progress (EP): Measures the distance traveled $D_{agent}$ relative to a safe upper bound $D_{ref}$ estimated by the PDM-Closed planner. Scores are discarded if $D_{ref}$ < 5 m. The score is computed as:

(11) $$EP=clip\left(\frac{D_{agent}}{D_{ref}},0,1\right)$$

Time to Collision (TTC): A binary safety metric based on the minimum time-to-collision value. The safety threshold is typically set to $\tau_{safe}$ = 1.0 s. The score is calculated as:

(12) $$TTC=\left\{\begin{array}{lll} 1 & if\underset{t\in [0,T]}{\min}({TTC}_{t})\geq \tau_{safe} & \\ 0 & otherwise & \end{array}\right.$$

Comfort (Comf): A binary metric validating if the trajectory’s kinematic properties remain within human-like comfort thresholds throughout the horizon. K represents the set of kinematic variables (acceleration, jerk, yaw rate) and $\theta_{k}$ are their corresponding thresholds. The score is calculated as:

(13) $$Comf=I\left(\forall t\in [0,T],\forall k\in K,|v_{k}(t)|\leq \theta_{k}\right)$$

On NAVSIM, HiPro-AD achieves strong performance for end-to-end planning. As shown in Table 4, under a camera-only setting our method outperforms prior approaches across all key metrics, reaching a PDMS of 92.6, which is more than four points higher than DiffusionDrive (88.1). Notably, the gains on DAC and EP highlight the advantages of the proposal-centric sparse paradigm for understanding complex road structure and making efficient driving decisions. Compared with multimodal methods such as VADV2 and Transfuser, HiPro-AD attains better results using only visual input, supporting the dual benefits of computational efficiency and planning effectiveness. The temporal attention mechanism yields smoother, more physically plausible trajectories, while the pairwise ranking-based scorer supports stable selection of the best candidate from multiple proposals.

### 4.2. Bench2Drive Benchmark

To simulate realistic driving scenarios and evaluate the closed-loop performance of our model, we conducted experiments on the CARLA [40] simulation platform using the Bench2Drive [41] benchmark. Bench2Drive is a large-scale benchmark designed for the comprehensive assessment of end-to-end autonomous driving systems. Its official training set comprises approximately 2 million fully annotated frames derived from over 10,000 short video clips, covering 44 diverse interactive scenarios (e.g., cut-ins, overtaking, and bypassing), 23 weather conditions, and 12 distinct towns to ensure extensive environmental diversity.

A key feature of Bench2Drive is its short-route closed-loop evaluation protocol. The benchmark defines 220 routes, each approximately 150 m in length, where every route targets a specific safety-critical interaction. This design effectively reduces the high variance typically associated with long-route evaluations and enables fine-grained, independent assessment of five advanced driving skills: lane merging, overtaking, yielding, traffic sign recognition, and emergency braking. For fair comparison, we utilized the standardized base subset (1000 clips), with 950 used for training and 50 for open-loop validation.

The evaluation metrics are both comprehensive and rigorous. Open-loop performance is measured by the average L2 distance between the planned and expert trajectories. Closed-loop performance is assessed via four core metrics, mathematically formulated as follows:

Success Rate (SR): The percentage of routes completed safely without collisions or traffic infractions within the time limit.

(14) $$SR=\frac{N_{success}}{N_{total}}\times 100\%$$

Driving Score (DS): A composite metric weighting route completion against infractions. For the *i*-th route, let $R_{i}\in [0,1]$ be the completion ratio and $P_{i}\in [0,1]$ be the penalty factor derived from infractions.

(15) $$DS=\frac{1}{N_{total}}\sum_{i=1}^{N_{total}}(R_{i}\times P_{i})\times 100\%$$

Efficiency: Measures the ego vehicle’s ability to maintain traffic flow, defined as the ratio of its average speed ${\overset{-}{v}}_{ego}$ to that of surrounding traffic ${\overset{-}{v}}_{traffic}$.

(16) $$Efficiency=\frac{{\overset{-}{v}}_{ego}}{{\overset{-}{v}}_{traffic}}\times 100\%$$

Comfort (Comf): Defined as the ratio of smooth trajectory segments to the total number of segments $S_{total}$. A segment is deemed smooth if its kinematic parameters (lateral acceleration, yaw rate, and jerk) remain within expert thresholds Θ.

(17) $$Comf=\frac{1}{S_{total}}\sum_{s=1}^{S_{total}}I({kinematics}_{s}\leq \Theta )\times 100\%$$

As presented in Table 5, our method demonstrates highly competitive performance on the rigorous Bench2Drive benchmark. Notably, HiPro-AD achieves superior results in both Success Rate and Driving Score without relying on expert teacher models or privileged information. This confirms the robustness of our proposal-centric sparse paradigm in handling complex dynamic interactions. Moreover, owing to the lightweight network design, our system significantly reduces inference latency to 67 ms, demonstrating high computational efficiency and real-time applicability.

### 4.3. Ablation Studies

To assess the contribution of individual components, we conduct ablations on the NAVSIM benchmark. The study systematically evaluates key elements of HiPro-AD by progressively introducing the improved IM-ResNet-34 feature extractor and the STFormer sparse planning paradigm, and analyzing their effects on planning performance.

As presented in Table 6, with the baseline configuration, the composite metric PDMS is 78.5; NC and DAC reach 97.6 and 93.0, respectively, but EP is relatively low (68.9), indicating limited adaptability in complex scenarios. Introducing IM-ResNet-34 alone raises PDMS to 83.6, with improvements across all metrics—most notably EP increases from 68.9 to 77.5—highlighting the benefits of depthwise separable convolutions and channel attention for efficient, high-quality feature extraction. Adding STFormer on top of IM-ResNet-34 further lifts PDMS to 89.4, with DAC and EP improving to 97.2 and 86.2, demonstrating the effectiveness of temporal attention and the proposal-centric sparse paradigm for capturing trajectory dynamics and improving smoothness.

Finally, combining both with the pairwise scorer yields the best results: PDMS 92.6, with NC, DAC, TTC, and EP all substantially improved. These results indicate strong synergy between IM-ResNet-34 and STFormer, jointly enhancing robustness and planning accuracy in dynamic environments. Overall, the ablations confirm the necessity of each component and show that their combination maximizes performance gains, providing empirical support for the efficiency of HiPro-AD.

**Figure 6:**
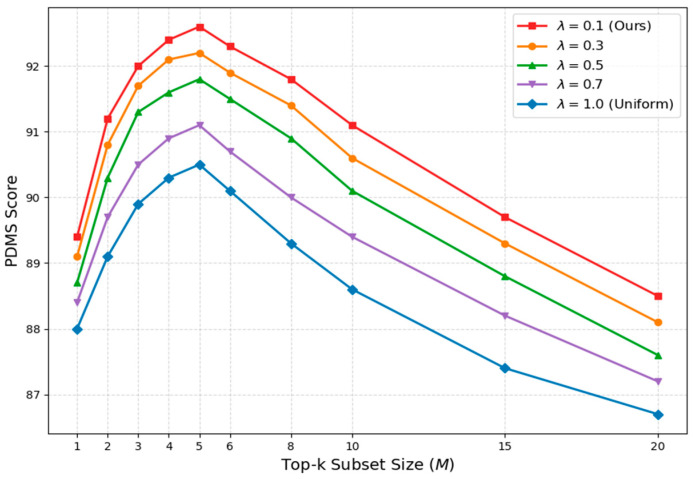
Sensitivity analysis of loss hyperparameters.

To strictly validate the architectural design of our Top-k Multi-Modal Loss, we conducted a comprehensive sensitivity analysis regarding the subset size *M* and the discount factor λ. As illustrated in Figure 6, we assessed the Planning Decision Metric Score across a detailed range of subset sizes from 1 to 20 under various discount schedules. The findings demonstrate a significant performance stratification governed by the discount factor, where our proposed coarse-to-fine strategy with a factor of 0.1 consistently achieves superior scores compared to the uniform supervision baseline. This suggests that applying looser constraints during early iterations facilitates more effective exploration of the solution space. Concurrently, the subset size *M* exhibits a clear inverted U-shaped trajectory. Increasing the subset size from 1 to 5 enhances performance by capturing multimodal diversity, whereas extending beyond this peak causes a steady decline. This downward trend corroborates the theoretical insight that optimizing an excessive number of candidates introduces detrimental gradient noise from low-quality proposals. Therefore, we identify the combination of a subset size of 5 and a discount factor of 0.1 as the robust optimal setting.

**Figure 7:**
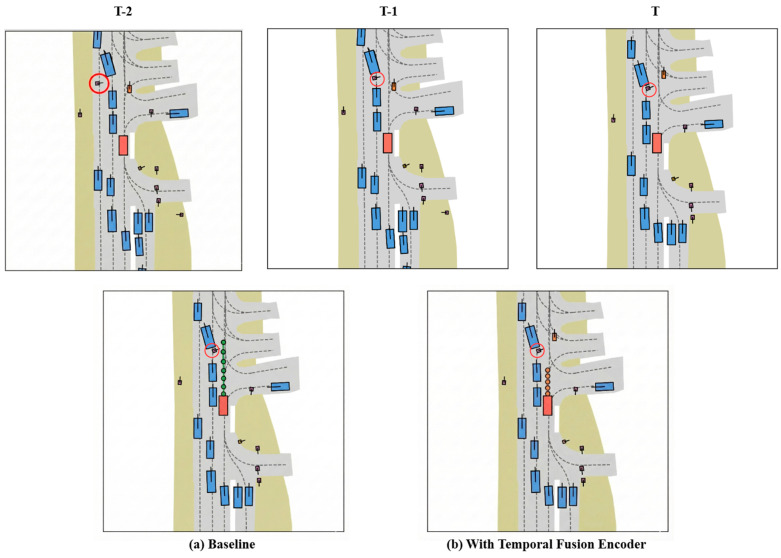
Qualitative ablation study on robustness against dynamic occlusion. The top row displays the temporal sequence of the scenario, in which a pedestrian highlighted by a red circle is visible at T-2 s but becomes occluded by an adjacent vehicle at T-1 s and T s. The bottom row compares the planning results at the current frame T. (**a**) Baseline: Without the Temporal Fusion Encoder, the model fails to recall the occluded pedestrian and plans a risky, aggressive trajectory represented by the green dots. (**b**) With Temporal Fusion Encoder: With temporal fusion, the model utilizes historical context to infer the presence of the pedestrian, generating a safe, yielding trajectory indicated by the orange dots.

To intuitively demonstrate the efficacy of the Temporal Fusion Encoder (TFE) in handling occlusions, we present a comparative case study in Figure 7. The scenario illustrates a typical “ghost probe” situation where a pedestrian is momentarily visible but subsequently enters a blind spot. As depicted in the temporal sequence in the top row, the pedestrian highlighted by the red circle is visible at T-2 but becomes completely occluded by a roadside vehicle at the subsequent T-1 and T timestamps.

The Baseline model, as illustrated in Figure 7a, lacks historical memory capabilities and consequently fails to account for the occluded pedestrian. It erroneously identifies the lane as clear and generates an aggressive trajectory, represented by the green dots, which poses a high collision risk. In contrast, Figure 7b demonstrates the efficacy of the HiPro-AD framework equipped with the Temporal Fusion Encoder (TFE). By successfully retaining the spatial information of the pedestrian from T-2 within its BEV memory bank, our model leverages this temporal context to anticipate the potential hazard. Accordingly, it plans a defensive trajectory, indicated by the orange dots, allowing the vehicle to yield to the unseen pedestrian and thereby ensuring safety. These qualitative results strongly validate that our proposal-centric sparse paradigm effectively mitigates the adverse effects of sensor occlusions.

### 4.4. Qualitative Analysis

**Figure 8:**
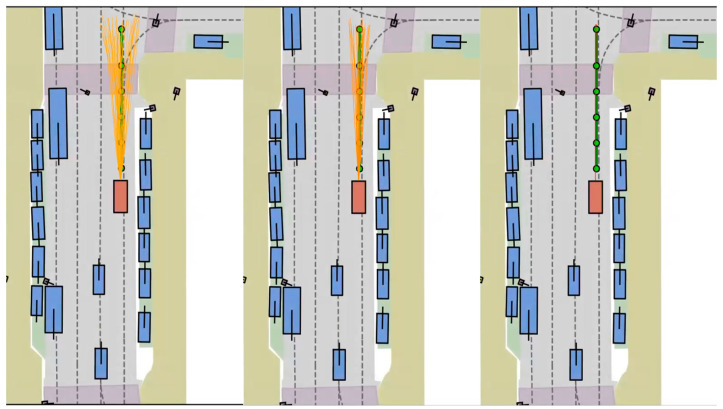
The Trajectory Refinement Process. The ground-truth human path is shown in green, and the model-generated proposals are shown in orange.

To visually demonstrate the effectiveness of our proposed method, we conducted extensive qualitative evaluations in the NAVSIM test environment. The results clearly indicate that our model not only generates safe and contextually valid trajectory proposals but, more critically, can iteratively refine its planned path to progressively approximate the behavior of a real human driver. As shown in Figure 8, we illustrate the model’s trajectory refinement process. In the figure, the green trajectory represents the ground-truth human trajectory, which serves as our evaluation standard. The cluster of orange trajectories represents the multiple proposals generated by our model in its initial stage. It is evident that while these initial proposals align with the target direction, they are quite dispersed, reflecting the model’s uncertainty across various possible paths during early planning. However, through our designed refinement module, the model evaluates the quality of these candidates and progressively optimizes its selection. The resulting trajectories become increasingly concentrated, closely approaching the green human trajectory in both shape and path. This process provides strong evidence that our method can effectively distill a broad possibility space and converge to a more precise and human-like driving decision.

**Figure 9:**
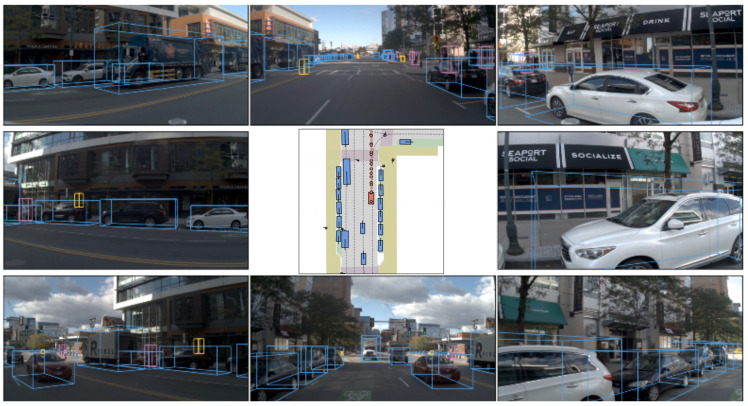
Visualization results in a NAVSIM scene.

We visualize the planning results of our method in another NAVSIM urban scene, as shown in Figure 9. The figure consists of two main parts: the central BEV illustrates the model’s environmental understanding and its final planning outcome. Surrounding the BEV are the camera images, which include the 3D object detection bounding boxes for other dynamic obstacles in the scene.

**Figure 10:**
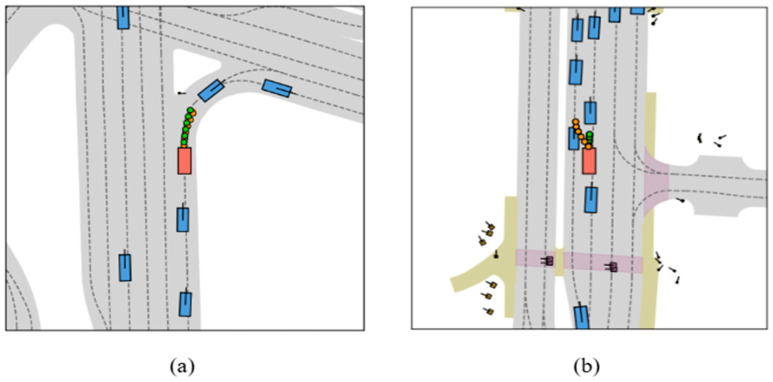
Comparative visualization of success and failure cases. The green curve indicates the ground-truth trajectory and the orange curve represents the path planned by our method. Subfigure (**a**) displays a successful unprotected turn where the model correctly handles the intersection geometry. Subfigure (**b**) illustrates a failure case involving a collision risk during a lane change in dense traffic.

To rigorously delineate the operational capabilities of our framework, we analyze the representative cases shown in Figure 10. The successful scenario in Figure 10a validates the proficiency of the model in executing complex maneuvers where the planned path aligns seamlessly with the expert trajectory during an unprotected turn. In contrast, the failure mode depicted in Figure 10b highlights current limitations in high-density traffic. Here the planner fails to secure a safe gap during a lane change amidst aggressive interference from surrounding vehicles which leads to a collision risk. This comparison clarifies that while the sparse paradigm provides robust structural navigation handling extreme interactive density remains a direction for future enhancement.

## 5. Conclusions

In this paper, we propose HiPro-AD, a novel end-to-end autonomous driving planning framework designed to address the challenges of high computational cost and limited interpretability in existing methods. By introducing a proposal-centric sparse paradigm, HiPro-AD effectively positions the planning task at the core of the perception-decision pipeline, thereby eliminating the reliance on resource-intensive dense BEV representations. Our efficiency-oriented IM-ResNet-34 network significantly reduces computational overhead while preserving feature quality. Furthermore, the core STFormer module leverages a Temporal Fusion Encoder to model temporal dynamics for smooth, physically plausible planning, while proposal-anchored spatial cross-attention enables the precise fusion of multi-view features. To further enhance decision-making, a pairwise ranking scorer is employed to accurately select the optimal trajectory from diverse candidates. Extensive experiments on the NAVSIM and Bench2Drive benchmarks demonstrate the superior performance of HiPro-AD compared to existing dense BEV paradigms using only camera input. Ablation studies confirm the synergistic effectiveness of these key components, and qualitative analyses illustrate the model’s capability to iteratively refine dispersed proposals into human-like driving behaviors. In summary, HiPro-AD offers an efficient, robust, and interpretable solution for scalable end-to-end autonomous driving.

## Figures and Tables

**Table 1:** Comparison of Learning Paradigms in Autonomous Driving.

Learning Mode	Representative Methods	Advantages	Limitations
Imitation Learning (IL)	UniAD/VAD	Unified Optimization: Jointly optimizes perception, prediction, and planning to mitigate cascading errors and information loss.	Resource Intensity: High computational complexity and inference latency hinder real-time deployment on edge devices.
	TransFuser [13]	Data Scalability: Directly utilizes large-scale expert demonstrations.	Causal Confusion: Prone to learning spurious correlations (e.g., background bias).
Reinforcement Learning (RL)	PPO [9]	Long-horizon Planning: Optimizes for long-term cumulative rewards.	Sample Inefficiency: Requires extensive interactions for convergence.
	SAC [10]	Super-human Potential: Explores novel strategies without reliance on human labels.	Reality Gap: Difficult to transfer simulation-trained policies to the real world safely.
Knowledge Distillation	Roach [11]	Feature Enhancement: Student models acquire robust representations from privileged teachers.	Pipeline Complexity: Involves a convoluted multi-stage training protocol.
	TCP [14]	Inference Efficiency: Achieves high performance with limited sensor inputs.	Oracle Dependency: Strictly relies on ground-truth states available only in simulators.
World Models	MILE [12]	Spatiotemporal Modeling: Deep understanding of scene dynamics and future states.	Computational Cost: High resource demands for both training and inference.
	DriveDreamer [15]	Self-Supervision: Learns from massive unlabeled video data.	Physical Inconsistency: Risk of generative hallucinations that may violate physical laws or geometric constraints.

**Table 2:** Comparison of attention mechanisms and their suitability for the proposed framework.

Attention Mechanism	Properties	Adoption	Rationale
SENet/GSoP-Net [24,25]	Global pooling; 2nd-order stats	No	Inefficient: High computational cost or parameter overhead compared to ECA.
ECA-Net [26]	Local 1D cross-channel interaction	Yes	Efficient: Enhances channel semantics with negligible overhead; ideal for our lightweight encoder.
STN [27]	Explicit spatial transformation	No	Rigid: Limited flexibility compared to modern deformable sampling.
Non-Local/ViT [28,29]	Global dense self-attention	No	High Latency: Quadratic complexity $O(N^{2})$ on dense grids makes real-time planning infeasible.
Swin Transformer [30]	Hierarchical window-based attention	No	Dense: Still processes dense regions; incompatible with our proposal-centric sparse paradigm.
Deformable self-attention	Sparse adaptive point sampling	Yes	Sparse: Focuses computation strictly on trajectory proposals, ignoring irrelevant background.
CBAM/BAM [31,32]	Serial/Parallel channel-spatial fusion	No	Redundant: Complex multi-branch designs increase latency without proportional gains for our task.
Triplet Attention [33]	Cross-dimension	No	Redundant: Our proposal anchors and PE already explicitly model geometry and position.

**Table 3:** Hyper-parameters.

Hyper—Parameter	Value
Proposal number N	64
Iteration number K	4
Planning time step interval	0.5s
Channel dimension C	256
Hidden size	256
Feed—forward size	1024
Pillar reference point number Nref	4
Proposal loss discount λ	0.1
NAVSIM future planning horizon T	8
NAVSIM image input down-sample rate	0.4

**Table 4:** Open-loop Results with Closed-loop Metrics on NAVSIM Benchmark.

Method	Input	NC	DAC	TTC	Comf.	EP	PDMS
PDM-Closed [36] (Rule-based)	Perception GT	94.6	99.8	86.9	99.9	89.9	89.1
VADV2-V8192 [37]	Camera & Lidar	97.2	89.1	91.6	100	76.0	80.9
Transfuser [13]	Camera & Lidar	97.7	92.8	92.8	100	79.2	84.0
DRAMA [38]	Camera & Lidar	98.0	93.1	94.8	100	80.1	85.5
DiffusionDrive [8]	Camera & Lidar	98.2	96.2	94.7	100	82.2	88.1
UniAD [6]	Camera	97.8	91.9	92.9	100	78.8	83.4
PARA-Drive [39]	Camera	97.9	92.4	93.0	99.8	79.3	84.0
HiPro-AD (Ours)	Camera	98.6	98.7	95.3	100	89.2	92.6

**Table 5:** Open-loop and Closed-loop Results of E2E Methods on Bench2Drive Benchmark.

Method	Latency	Open-Loop	Closed-Loop			
		Avg. L2	Efficiency	Comfort	Success Rate (%)	Driving Score
AD-MLP [42]	4 ms	3.64	48.45	22.63	0.00	18.05
UniAD-Tiny [6]	445 ms	0.80	123.92	47.04	13.18	40.73
UniAD-Base [6]	558 ms	0.73	129.21	43.58	16.36	45.81
VAD [7]	359 ms	0.91	157.94	46.01	15.00	42.35
DriveTransformer [43]	212 ms	0.62	100.64	20.78	35.01	63.46
HiPro-AD (Ours)	67 ms	0.75	159.31	32.19	37.31	65.48

**Table 6:** Ablation Studies on the NAVSIM Benchmark.

Scene Encoder	Scene Encoder	Scorer	NC	DAC	TTC	EP	PDMS
ResNet-34	BEVFormer	BCE	97.6	93.0	92.9	68.9	78.5
IM-ResNet-34	BEVFormer	BCE	98.0	94.9	93.8	77.5	83.6
IM-ResNet-34	STFormer	BCE	98.4	97.2	94.8	86.2	89.4
IM-ResNet-34	STFormer	Pairwise	98.6	98.7	95.3	89.2	92.6

## Data Availability

The data presented in this study are available upon request from the corresponding author.
